# Analysis of insulin pump settings in children and adolescents with type 1 diabetes mellitus

**DOI:** 10.1186/1687-9856-2015-S1-P17

**Published:** 2015-04-28

**Authors:** Ning Lau Yu, Sophy Korula, Kristine Heels, Ines Krass, Geoffrey Ambler

**Affiliations:** 1 Department of Pharmacy, The University of Sydney, Sydney, NSW, Australia; 2 Institute of Diabetes and Endocrinology, The Children’s Hospital at Westmead, Sydney, NSW, Australia

## Aim

To characterise current insulin pump settings used in young patients with type 1 diabetes mellitus (T1DM) and their relationship to glycaemic and weight control.

## Methods

This retrospective study included patients aged <18 years old with T1DM duration >1 year who were using the Medtronic pump device. Data from the insulin pumps including number of blood glucose (BG) tests per day, basal and bolus insulin parameters, carbohydrate ratio (CR) and insulin sensitivity factors (ISF) were averaged over 14 days for statistical analyses. Anthropometric data and recent HbA1c was also recorded.

## Results

292 patients (144 males, 148 females) were included in the study. Participants had a median age (IQR) of 12.9 (10.0-15.1) years and pump duration of 2.8 (1.5-4.2) years. No significant differences in median HbA1c (IQR) were observed in preschool (n=14; HbA1c (8.0% (7.0 to 8.0%)), prepubertal (n=105; HbA1c (8.0% (8.0 to 9.0%)) and adolescent subjects (n=173; HbA1c (8.0% (8.0 to 9.0%)). Adolescents took significantly fewer boluses and BG tests per day compared to younger children (p<0.05). Age specific diurnal variation in basal insulin doses was noted. Additionally, stronger carbohydrate cover was used in real-life compared to the theoretical 500 rule while weaker corrections were used in real-life compared to the 100 rule. Predictors of lower HbA1c values included higher number of daily boluses, greater number of blood glucose testing per day, lower average CR/500 rule ratio and higher average ISF/100 rule ratio adjusted for age (R2 =0.22; p<0.01). Predictors of favourable weight (lower BMI-SDS) included lower total daily dose(units/day), lower percentage of basal to total daily insulin dose, weaker average CR and higher number of different CR adjusted for age (R2 = 0.33; p<0.05), although age was the strongest predictor (older age associated with lower BMI-SDS).

## Conclusion

Insulin pump therapy requires continuous adjustments and glycaemic targets are achieved only by a minority. This study provided additional information on real life carbohydrate ratio and insulin sensitivity factor, which may be helpful in optimising pump therapy in the future.

